# After Them

**Published:** 1898-06

**Authors:** 


After Them.—The Cherokee County Medical Society has been actively seconding the work of the "Red Back" in exposing and getting after the holders of bogus diplomas. Dr. E. E. Guinn, the Secretary of that Society, has sent the Journal a copy of a circular which was published by order of the Society and mailed to the profession of the State.

The circular gives a statement from the *Chicago Tribune*, that the Attorney-General of Illinois had the notorious "Dr. Armstrong"—the old "medical" fraud "dragged into court" on a charge that the so-called "Independent Medical College" for which, the *Tribune* says, Armstrong obtained a charter on false representations, was "usurping the powers of a corporation," and asked that "Armstrong be required to show cause why this charter should not be revoked," etc.

In the circular referred to, sent out by our friends in Cherokee County, the entire correspondence is given, verbatim, as it occurred between the "President"—Armstrong and an illiterate hostler somewhere in Delaware. The result of the correspondence was the granting of the hostler a "diploma" *in absentia*, which was sent for a consideration of $25—by express—C. O. D.

Dr. Guinn adds that by the activity of the County Society they had ridded the section of one or two holders of these bogus diplomas.

The JOURNAL has, in former issues, so thoroughly ventilated this matter that it is unnecessary to go into further particulars. We are much gratified to see a County Society taking hold of the case.

As we have no law, or, rather a law which is worse than no law in that it recognizes as *license to practice* any kind of diploma from anywhere, or any kind of a certificate from any kind of board or alleged board, and the clerk of the court is the sole judge of said diplomas and certificates (and it is all fish that comes to his net—everything that he records is money in *his* pocket—why—should *he* discriminate—even if he were able?)—the only recourse left the medical profession for their own protection is to boldly prosecute all holders of bogus documents, enforce the law, as poor as it is. The JOURNAL sends greeting to the Cherokee County doctors and bids them persevere in the good work.

